# Integration of molecular features with clinical information for predicting outcomes for neuroblastoma patients

**DOI:** 10.1186/s13062-019-0244-y

**Published:** 2019-08-23

**Authors:** Yatong Han, Xiufen Ye, Chao Wang, Yusong Liu, Siyuan Zhang, Weixing Feng, Kun Huang, Jie Zhang

**Affiliations:** 1 Department of Automation, Harbin Engineering University, Harbin, China; 2 Department of Neurosurgery, Stanford University, California, USA; 3 Thermo Fisher Scientific, Waltham, MA USA; 4 Department of Medicine, Indiana University School of Medicine, Indianapolis, USA; 5 Department of Medical and Molecular Genetics, Indiana University School of Medicine, Indianapolis, USA

**Keywords:** Neuroblastoma prognosis, Survival time prediction, Gene co-expression network, Consensus clustering, lmQCM

## Abstract

**Background:**

Neuroblastoma is one of the most common types of pediatric cancer. In current neuroblastoma prognosis, patients can be stratified into high- and low-risk groups. Generally, more than 90% of the patients in the low-risk group will survive, while less than 50% for those with the high-risk disease will survive. Since the so-called “high-risk” patients still contain patients with mixed good and poor outcomes, more refined stratification needs to be established so that for the patients with poor outcome, they can receive prompt and individualized treatment to improve their long-term survival rate, while the patients with good outcome can avoid unnecessary over treatment.

**Methods:**

We first mined co-expressed gene modules from microarray and RNA-seq data of neuroblastoma samples using the weighted network mining algorithm lmQCM, and summarize the resulted modules into eigengenes. Then patient similarity weight matrix was constructed with module eigengenes using two different approaches. At the last step, a consensus clustering method called Molecular Regularized Consensus Patient Stratification (MRCPS) was applied to aggregate both clinical information (clinical stage and clinical risk level) and multiple eigengene data for refined patient stratification.

**Results:**

The integrative method MRCPS demonstrated superior performance to clinical staging or transcriptomic features alone for the NB cohort stratification. It successfully identified the worst prognosis group from the clinical high-risk group, with less than 40% survived in the first 50 months of diagnosis. It also identified highly differentially expressed genes between best prognosis group and worst prognosis group, which can be potential gene biomarkers for clinical testing.

**Conclusions:**

To address the need for better prognosis and facilitate personalized treatment on neuroblastoma, we modified the recently developed bioinformatics workflow MRCPS for refined patient prognosis. It integrates clinical information and molecular features such as gene co-expression for prognosis. This clustering workflow is flexible, allowing the integration of both categorical and numerical data. The results demonstrate the power of survival prognosis with this integrative analysis workflow, with superior prognostic performance to only using transcriptomic data or clinical staging/risk information alone.

**Reviewers:**

This article was reviewed by Lan Hu, Haibo Liu, Julie Zhu and Aleksandra Gruca.

**Electronic supplementary material:**

The online version of this article (10.1186/s13062-019-0244-y) contains supplementary material, which is available to authorized users.

## Background

Neuroblastoma (NB) is one of the most common types of pediatric cancer, with patients being mostly children of age five or younger. It is a heterogeneous disease affecting different areas of the body, and the likelihood of cure varies according to age at diagnosis, extent of disease, and tumor biology [1]. NB patients are usually stratified into low-risk and high-risk groups with more than 90% of patients survive in the low-risk group while only less than 50% for those with high-risk disease can be cured. Since the high-risk patients still contain patients with mixed good and poor outcomes, more refined stratification needs to be established to enable personalized treatment plan for the patients with worse outcomes, whereas patients with better prognosis can avoid unnecessary over-treatment.

With the accumulation of large amount of clinical, genomic, and pathological data for NB, a potential approach to improve the prognosis can be achieved by integrating genetic mutations, gene expression profiles, tissue and organ morphological features as well as clinical phenotypes to make a holistic decision. To achieve this goal, new methods for integration of different modalities of data need to be developed. To address this, the consensus clustering method, which integrates multiple clustering results from different types of data for the same patient cohort to achieve a single clustering of the data, has been introduced for this purpose [2]. Currently there are two major approaches to perform the consensus learning: 1) probabilistic approach, which adopts a maximum likelihood formulation to generate the consensus clustering results using the Dirichlet mixture model given the distributions of base labels [3]; and 2) similarity approach, which directly finds consensus clusters that agree the most with the input base clusters [4]. Despite the quick development of this method, most of the consensus learning algorithms still cannot be directly applied to multi-modal data with mixed data types (e.g., numerical data for gene transcription levels and categorical data for clinical stages of the patients), which limits the clinical applications of this method. In this work, we present an effective and flexible data integration workflow for integrating numeric transcriptomic data and categorical clinical information based on our previously developed consensus clustering algorithm Molecular Regularized Consensus Patient Stratification (MRCPS) [5]. MRCPS has been successfully applied for predicting outcomes for triple negative breast cancers [5]. Our goal is to identify a consensus partition of patients from the combination of transcriptomic data and clinical features (i.e., clinical stage and risk level) to better refine NB prognosis.

**Fig. 1:**
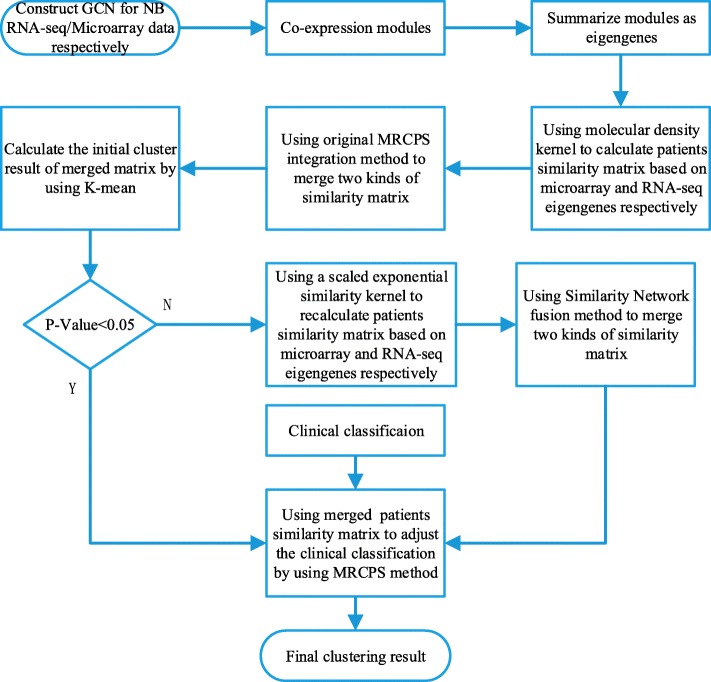
The workflow of integrating molecular features with clinical features for NB patient stratification

The integrated workflow of MRCPS is shown in Fig. 1. Our data were obtained from the Neuroblastoma Data Integration Challenge of CAMDA 2017. Since both RNA-seq and gene expression microarray data are available for this cohort, we took advantage of both data types, which is not required for this workflow per se. However, the sheer large number of features (i.e., gene transcripts and probesets) in the transcriptomic data poses a challenge on the downstream data integration as well as the statistical power for detecting representative gene expression features. To reduce the data dimensionality and improve the statistical power, we first applied our previously developed network mining algorithm lmQCM (local maximum Quasi-Clique Merger) to identify densely connected co-expressed gene modules [6] and summarized each module into an “eigengene” using the protocol described in [7]. The identified co-expression modules not only reduce the data dimension, but often contain strong signals for important biological processes, functions, or copy number variants associated with the modules, which facilitates the downstream integration with other data types and interpretation of the results. Next, we applied MRCPS method to combine the eigengenes, clinical stage, and risk level information. The intuition for MRCPS is that each data type leads to a patient network and the goal of the algorithm is to regularize the patient network formed by clinical stage classification using a weight matrix generated from molecular data. This weight matrix defines the affinity between patient samples in the molecular features space. It can be derived from molecular subtypes and estimation of density-based models. However, the original MRCPS method is sensitive to the classification result of the molecule features, it may impact the integration results negatively if the classification by the molecule features is not robust enough. Therefore in this paper, we took two approaches to generate weighted patient similarity matrix from transcriptomic data and integrated it with categorical clinical features from the same patient cohort and pursued a consensus clustering of the cohort. Specifically, in the cases that the initial molecular feature clustering failed to stratify patients into significant survival groups (i.e., log-rank test *p*-value > 0.05), we switch to a patient similarity matrix based on a graph method to integrate molecular data with clinical stage and risk level information. Using this strategy, we were able to further stratify the high-risk patients into subgroups with significantly different survival times superior to using clinical stage. The associated co-expression gene features also confirmed previous findings with known NB genes [8].

## Methods

### Dataset and preprocessing

The data used in this study was obtained from the Neuroblastoma Data Integration Challenge of CAMDA 2017, which is also available in NCBI Gene Expression Omnibus as GSE47792 [9]. It contains tumor samples of 498 neuroblastoma patients from seven countries: Belgium (*n* = 1), Germany (*n* = 420), Israel (*n* = 11), Italy (*n* = 5), Spain (*n* = 14), United Kingdom (n = 5), and United States (n = 42). The patients’ age at diagnosis varied from 0 to 295.5 months (median age, 14.6 months).

Transcriptome datasets from both microarray (Agilent 44 K oligomicroarray) and RNA-seq (Illumina HiSeq 2000) platforms were obtained for the above 498 patients with known clinical endpoints. The RNA-seq data includes 60,788 transcripts while the microarray data includes 45,198 probesets, both from the same 498 primary neuroblastomas. Tumor stage was classified according to the International Neuroblastoma Staging System (INSS): stage 1 (*n* = 121), stage 2 (*n* = 78), stage 3 (*n* = 63), stage 4 (*n* = 183), and stage 4S (*n* = 53). 176 patients were labeled as high-risk, which defined as stage 4 disease for more than 18 months since diagnosis as well as patients of any age and stage with MYCN-amplified tumors [9]. For RNAs-seq data, processed FPKM values were downloaded which went through read mapping, gene expression quantification and normalization as described in [9]. We identified 9583 unique genes whose expression profiles are present in both RNA-seq and microarray datasets with matched gene symbols. To remove any further batch effect within a dataset, we further converted gene expression values into z-score values within each dataset for further gene co-expression network mining and data integration.

### Gene co-expression network mining and eigengene summarization

We applied our previously developed weighted network mining algorithm lmQCM [6] for gene co-expression module mining. Unlike the popular algorithm WGCNA that utilizes hierarchical clustering and does not allow overlaps between clusters [10], lmQCM allows genes to be shared among multiple gene modules, agreeing with the biological fact that genes often participate in multiple biological processes. In addition, we have shown that lmQCM can find co-expressed gene modules that are often associated with structural variations such as copy number variances (CNVs) in cancers. The lmQCM algorithm requires four parameters, namely γ, λ, *t*, and β. Among these parameters, γ is the most important parameter as it determines if a new module can be initiated by setting the weight threshold for the first edge of the module as a new subnetwork. *t* and λ determine an adaptive threshold for the density of the network, which the mining algorithm will stop when the threshold is reached. β specifies the threshold for overlap ratio between two modules. If the overlap ratio between two modules (defined as the ratio between the size of overlap and the size of the smaller module) is larger than β, the two modules are then merged into a larger one. In practice, we found that with γ = 0.80, *t* = 1, λ = 2, and β = 0.4, the algorithm yielded gene modules with reasonable sizes (less than 500 genes).

In our analysis, we first computed the Spearman correlation coefficients between expression profiles of any pair of genes, then transform it into edge weight using a weight-normalization procedure adopted from spectral clustering in [11]. We mined co-expression modules separately in microarray and RNA-seq data. As the result, it identified 38 co-expressed gene modules for the microarray data and 24 modules for the RNA-seq data. The module gene expression levels were summarized into “eigengene” values using Principle Component Analysis (PCA) with the first principle component being the eigengene value for a specific module. They are used as the transcriptomic features for the survival prognosis.

### Molecular regularized consensus patient stratification (MRCPS)

We previously developed a mathematical formulation for integrative clustering of multiple-modal data. Specifically, we introduced a consensus clustering method called Molecular Regularized Consensus Patient Stratification (MRCPS) based on an optimization process with regularization [5]. This consensus clustering workflow is flexible, allowing integration of both categorical and numerical data. Due to the fact that the original MRCPS is sensitive to the initial result of molecular clustering, we developed two methods to build the patient similarity matrix using molecular density function and the similarity network fusion method as described below, to ensure the effectiveness of our consensus cluster method. They are the following:

#### Patient similarity weight matrix based on molecular density function

Cluster density function [12]: Based on the molecular features, a clustering algorithm such as K-means can be applied thus each patient *i* is clustered in its molecular subgroup. Then, we can define a cluster density function *f*(∙) for this sample. A typical choice of the density function is the Gaussian Kernel density function [9]:

1 $$ f(i)=\frac{1}{h^p{N}_i}{\sum}_{j=1}^{N_i}{K}_h\left({x}_i-{x}_j\right)=\frac{1}{N_i{\left(2\pi {h}^2\right)}^{\frac{p}{2}}}{\sum}_{j=1}^{N_i}\mathit{\exp}\left(-\frac{\left\Vert {x}_i-{x}_j\right\Vert }{2{h}^2}\right) $$

where *N*_*i*_ is the number of patients in the same cluster with features *x*_*i*_ ∈ *ℜ*^*p*^ and the summation enumerates over all the *N*_*i*_ patients in the cluster with *i*. Furthermore, and *K*_*h*_ is a Gaussian Kernel function with parameters *h*.

Then given two patients *i* and *j*, the “molecular affinity” between them can be defined as weight *W(i,j)* such that:

2 $$ W\left(i,j\right)=\left\{\begin{array}{c}f(i)\times f(j)\  if\ i\ne j\  and\ i,j\  are\  in\ the\ same\ cluster\\ {}0\kern3.00em \ \kern1em if\ i\ne j\  and\ i,j\  are\  in\ the\ different\ cluster\\ {}1\kern4.00em \  if\ i=j\end{array}\right. $$

#### Patient similarity weight matrix using a scaled exponential similarity kernel

In the cases that the initial clustering using the above matrix leads to a stratification of the patients without significant difference in survival times (i.e., log-rank test *p*-value > 0.05), we define another similarity weight matrix based on graph method, or a patient similarity network. Edge weights are represented by an n x n similarity matrix *W* with *W(i,j)* indicating the similarity between patients *d*_*i*_ and *d*_*j*_. *W(i,j)* is generated by applying a scaled exponential similarity kernel on the Euclidean distance *d (x*_*i*_*,x*_*j*_*)* between the patient features x_i_ and *x*_*j*_ [8].

3 $$ W\left(i,j\right)=\mathit{\exp}\left(-\frac{d^2\left({x}_i,{x}_j\right)}{\mu {\varepsilon}_{i,j}}\right) $$

where

4 $$ {\epsilon}_{i,j}=\frac{mean\left(d\left({x}_i,D(i)\right)+ mean\right(d\left({x}_j,D(j)\right)+d\left({x}_i,{x}_j\right)}{3} $$

Here D(*i*) is the cluster containing patient *i* and *mean*(*d*(*x*_*i*_, *D*(*i*)) is the average of Euclidean distance between *x*_*i*_.

Through the above method we obtain the patient similarity weight matrices from microarray and RNA-seq datasets respectively. They can be integrated using the following two approaches:

#### Original MRCPS integration method

The original MRCPS method is focused on the density in the overlap samples of same clusters of both the microarray and RNA-seq. The other density weight will be 0. The integrated density weight matrices as follows:

5 $$ {W}^{\ast}\left(i,j\right)=\sqrt{W^{(1)}\left(i,j\right)\circ {W}^{(2)}\left(i,j\right)} $$

where W^(1)^ is for microarray data and W^(2)^ for RNA-seq data.

#### Similarity network fusion (SNF)

This method was developed in the [13] to integrate data from multiple sources. In our work, we have two patient similarity weight matrices (m = 2). The key step of SNF is to iteratively update similarity weight matrix corresponding to each of the data types as follows:

6 $$ {\overset{\sim }{W}}_{t+1}^{(1)}={S}^{(1)}\times {W}_t^{\left(\overset{\sim }{2}\right)}\times {S^{(1)}}^T $$

7 $$ {\overset{\sim }{W}}_{t+1}^{(2)}={S}^{(2)}\times {W_t}^{\left(\overset{\sim }{1}\right)}\times {S^{(2)}}^T $$

Where ${W}^{\left(\overset{\sim }{m}\right)}$ is defined as:

8 $$ {W}^{\left(\overset{\sim }{m}\right)}=\left\{\begin{array}{c}\frac{W_{i,j}^{(m)}}{2{\sum}_{k\ne i}{W}_{i,k}^{(m)}}\  if\ i\ne j\\ {}\frac{1}{2}\  if\ i=j\end{array}\right. $$

Let *D(i)* represent a set of *x*_*i*_’s neighbors including *x*_*i*_ in *G*. Given a graph, *G*, we use K nearest neighbors (KNN) to measure local affinity. So *S*^*(m)*^ is defined as:

9 $$ {S}_{i,j}^{(m)}=\left\{\begin{array}{c}\frac{W_{i,j}^{(m)}}{2{\sum}_{k\in {N}_i}{W}_{i,k}^{(m)}}\  if\ i\ne j\\ {}0\  if\ i=j\ \end{array}\right. $$

That ${W}^{\left(\overset{\frown }{m}\right)}$ carries the full information about the similarity of each patient to all other patients whereas *S*^(m)^ only encodes the similarity to the K most similar patients for each patient. This procedure updates the weight matrices each time generating two parallel interchanging diffusion processes. After t steps, the overall weight matrix is computed

10 $$ {W}^{\ast}\left(i,j\right)=\frac{{\overset{\sim }{W}}_t^{(1)}\left(i,j\right)+{\overset{\sim }{W}}_t^{(2)}\left(i,j\right)}{2} $$

### Categorical distance metric

In order to apply the weight matrix from transcriptomic data to refine the patient clusters defined by the clinical features, we first need to define a distance metric for the clinical similarity between a pair of samples. The categorical distance metric between two clinical clusters *C*^*l*^, *C* is

11 $$ dis\mathrm{t}\left({C}^l,C\right)={\sum}_{i<j}{\left[{S}_{ij}^l-{S}_{ij}\right]}^2 $$

where *S*^*l*^_*ij*_ = 1 if the patients *i* and *j* are in the same cluster, and otherwise is 0. Specifically, given a set of *L* clinical partitions (in this work, we use clinical stage and clinical risk), and dist (,) the symmetric difference distance metric, we wish to find an overall partition C*:

12 $$ {C}^{\ast }=\frac{1}{L}\mathit{\arg}\underset{C}{\mathit{\min}}{\sum}_{l=1}^L dist\left({C}^l,C\right) $$

Next, we take the weight matrix generated from the molecular data to adjust the clinical clusters. We weighed each pair of patient similarity *S*_*ij*_ based on the fused similarity weight matrix W for every i and *j*. The underlying rationale is that, if two patient samples are in a cluster of poor molecular clustering result, similarity between them should be low. Thus, a lower weight is given to leverage the high clinical similarity *S*_*ij*_. Now, we can get an equation as following:

13 $$ {S}^{\ast }=\frac{1}{L}\mathit{\arg}\underset{S}{\mathit{\min}}{\sum}_{i=1}^L{\sum}_{i<j}{w}_{i,j}{\left[{S}_{ij}^l-{S}_{ij}\right]}^2 $$

We can optimize the following cost function to find the optimal partition of patients:

14 $$ {\overset{\sim }{S}}^{\ast }=\mathit{\arg}\underset{S}{\mathit{\min}}{\left\Vert {\overset{\sim }{S}}^L-\overset{\sim }{S}\right\Vert}_F^2 $$

Where ${\overset{\sim }{S}}^L=\frac{1}{L}{\sum}_{l=1}^L\left({S}^l\circ \sqrt{W}\right)$ and $\overset{\sim }{S}=S\circ \sqrt{W}$ are the Hadamard products with weight matrix W. ‖.‖_*F*_ denotes the matrix Frobenius Norm. The detail of this optimal progress is shown in [5].

### Cluster number determination

We evaluate the effectiveness of clustering results using mutual information, which has been adopted in traditional consensus clustering methods [14]. The optimal consensus is expected to have the maximal mutual information with the base clustering, meaning that it shares the most information. Therefore, the final clustering number *k* can be determined by maximizing the following Normalized Mutual Information (NMI) with the original clustering result C:

15 $$ {\phi}^{(NMI)}\left({C}_f,C\right)=\frac{\sum_u^M\Big(H\left({C}_u\right)+H\left({C}_f\right)-H\left({C}_u,{C}_f\right)}{\sqrt{H\left({C}_u\right)H\left({C}_f\right)}} $$

Where *H (C*_*u*_*)* is the entropy associated with *u*-th base clustering, *H (*C_f_*)* is the entropy arising from the final clustering label and *H (C*_*u*_*,*C_f_*)* is the mutual information between two clustering results.

### Gene ontology and pathway over-representation analysis

Two online gene ontology and pathway enrichment tools ToppGene (http://toppgene.cchmc.org) developed by Cincinnati Children’s Hospital Medical Center [15] and DAVID Gene Functional Classification Tool (http://david.abcc.ncifcrf.gov) [16] were used for all of the module functional and pathway over-representation analysis. ToppGene not only performs enrichment analysis on standard gene ontology, it also incorporates more than 20 different sources including pathway databases, human and mouse phenotypes, NCBI PubMed, transcription factor binding sites, and drug information for a comprehensive enrichment analysis.

DAVID provides a comprehensive set of functional annotation tools for investigators to understand biological meaning behind large list of genes.

Both tools used the entire human protein-encoded genome as the background reference gene list for over-representation analysis. The gene ontology terms with adjusted enrichment *p* value < 0.05 were considered over-represented terms, and listed for the genes in a specific module in the Results and the Additional file 1 and Additional file 4.

### Differential gene expression analysis

Differential gene expression analysis was performed on RNA-seq data between the subgroups of patients with the best prognosis and the worst prognosis (Group 4 and Group 5 respectively of Fig. 5(d)). The gene expression values of FPKM were first log-transformed to test and ensure for distribution normality, then the Student t-test was performed and the cutoff of 1.5 for the absolute value of foldchange as well as the adjusted *p*-value < 0.001 were used for differential expression.

## Results

### Improved NB prognosis by integrated MRCPS method over clinical stage or transcriptomic features alone, which identified a new prognosis group with worst outcomes

As shown in Fig. 1 of the MRCPS workflow, we applied two approaches to generate the patient similarity matrix of the molecular feature. Frist by using the cluster density function, and second by using the scaled exponential similarity kernel as described in the previous section. We then integrated molecular data with the patient classification information.

**Fig. 2:**
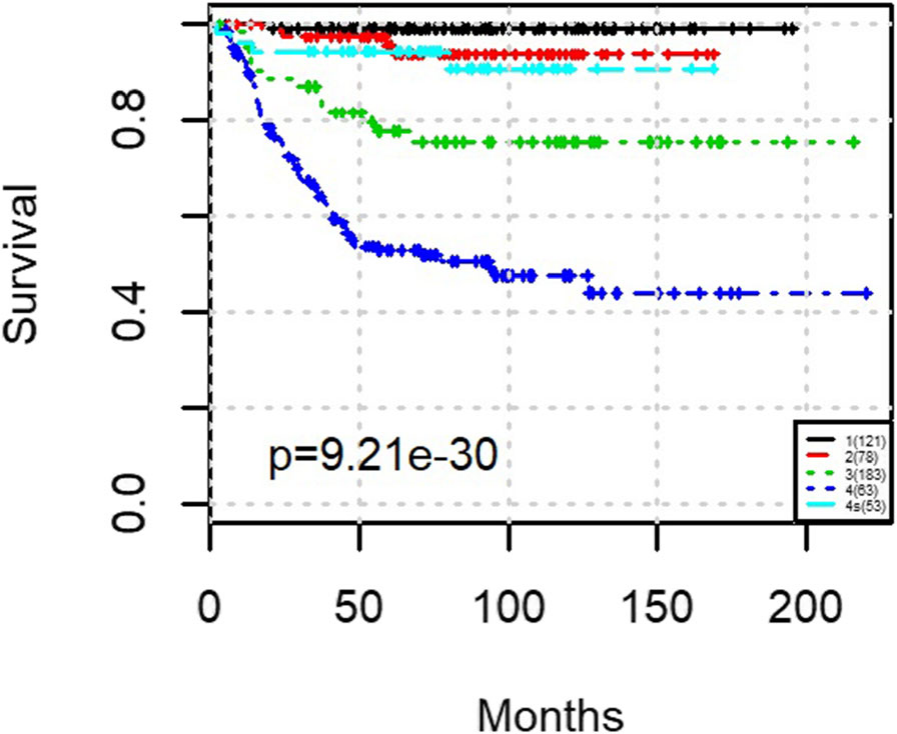
The Kaplan-Meier survival plot for the entire NB cohort using clinical stage information

**Fig. 3:**
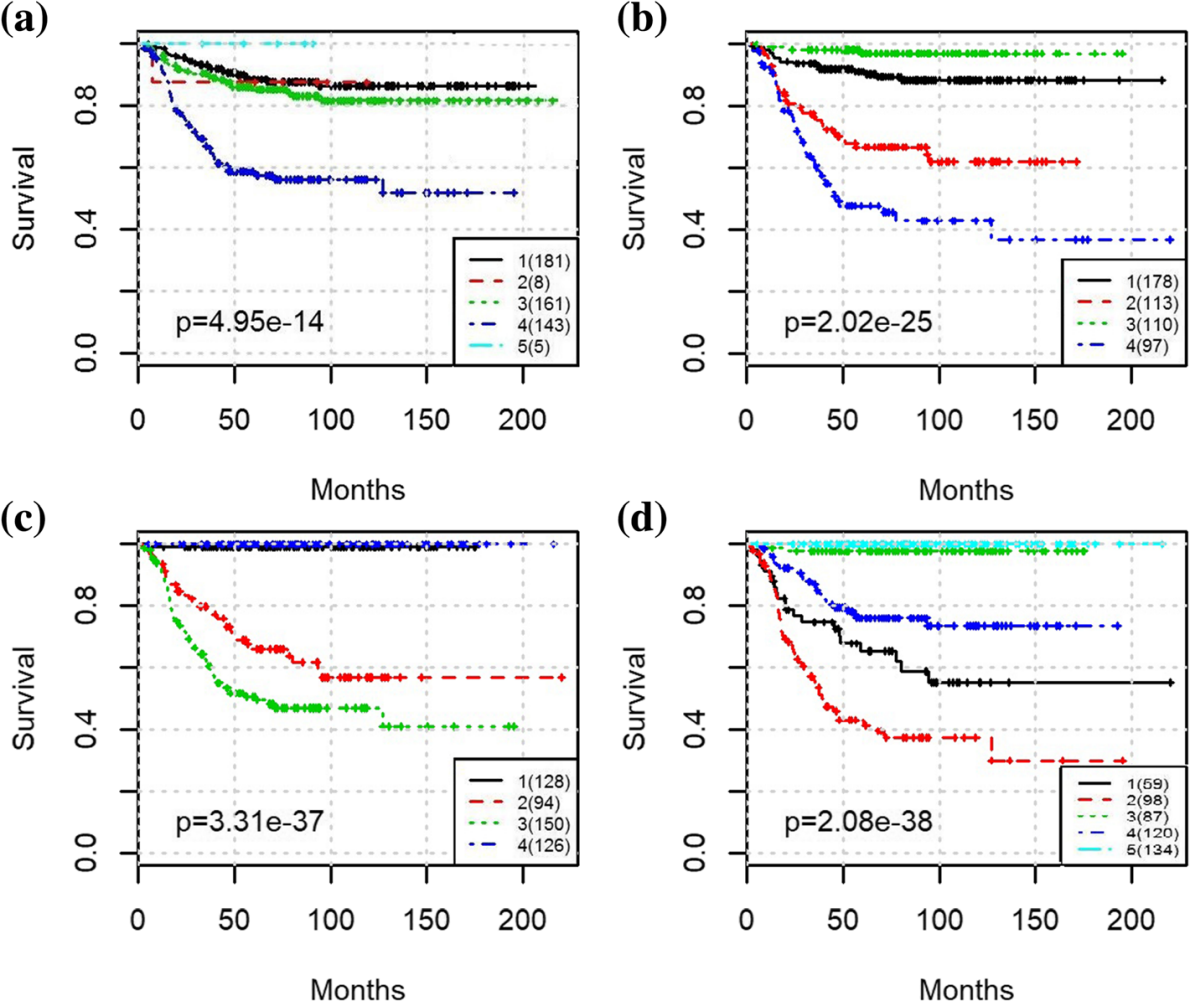
The Kaplan-Meier survival plot for the entire NB cohort with MRCPS of molecular density weight matrix: (a) Results from K-means clustering using only transcriptomic features; (b) Results from MRCPS of molecular density kernel integrated with clinical stage; (c) Results from MRCPS of molecular density kernel integrated with risk-level; (d) Results from MRCPS of molecular density kernel integrated with clinical stage and risk-level

**Fig. 4:**
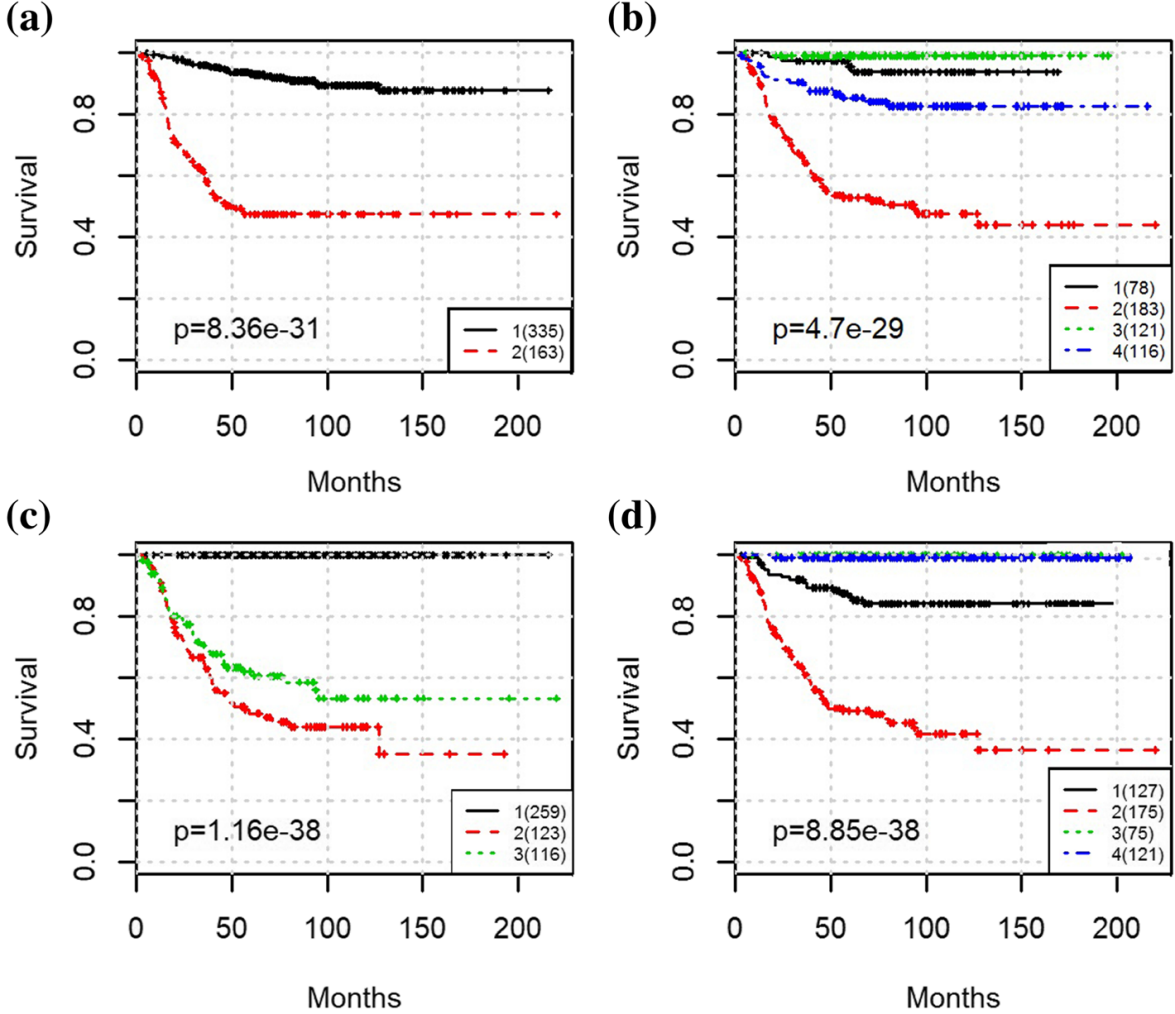
The Kaplan-Meier survival plot for the entire NB cohort with MRCPS of molecular similarity weight matrix. (a) Results from SNF using only transcriptomic features; (b) Results from MRCPS of scaled exponential similarity kernel integrated with clinical stage; (c) Results from MRCPS of scaled exponential similarity kernel integrated with risk-level; (d) Results from MRCPS of scaled exponential similarity kernel integrated with clinical stage and risk-level

To evaluate the prognostic performance of various methods, Kaplan-Meier survival curves were generated, and log-rank test between patients in different groups was applied. The Kaplan-Meier curve along with the *p* values for log-rank test from clinical staging is shown in Fig. 2. The MRCPS results using cluster density function are shown in Fig. 3, and the ones with scaled exponential similarity kernel are shown in Fig. 4.

For each approach, we also compared the classification results with those obtained using transcriptomic features alone (i.e., eigengenes from co-expression module mining). We used K-means (Fig. 3(a)) and the similarity network fusion (SNF) algorithm [9] (Fig. 4(a)) for transcriptomic features alone, which means only the clustering on molecular data of MRCPS of was used in this case.

As shown in Fig. 2, the clinical staging information separates patients into five groups (stages 1,2,3,4 s,4) with significantly different survival times (*p*-values for log-rank test was 9.21e-30). The prognostic results of using transcriptomic features (eigengenes) alone are shown in Figs. 3(a) and 4(a) respectively. While the patients can be well separated using transcriptomic feature alone, the prediction is inferior to the ones using clinical stage, suggesting that integrating clinical stage and risk level information may bring additional information to survival prediction. As expected, both molecular weight matrices from MRCPS generate better prognosis prediction than using clinical stage or transcriptomic feature alone, as shown in Figs. 3(d) and 4(c) (with log-rank p-values of 2.08e-3 and 1.16e-38, respectively). After integrating both the clinical stage and the risk factor, another intermediate survival group is identified (Fig. 3(d) Group 4). A closer examination of the patient groups shows a substantial overlap between the groups of Fig. 3(c) and Fig. 3(d): 84% Patients in group 3 and 5 from Fig. 3(d) overlap with the patients in group 1 and 4 from Fig. 3(c) (for details of the patient grouping please see the Additional file 2). As shown in the clustering results, MRCPS makes full use of clinical features and has the superior capability to cluster patients with significantly different outcomes.

**Fig. 5:**
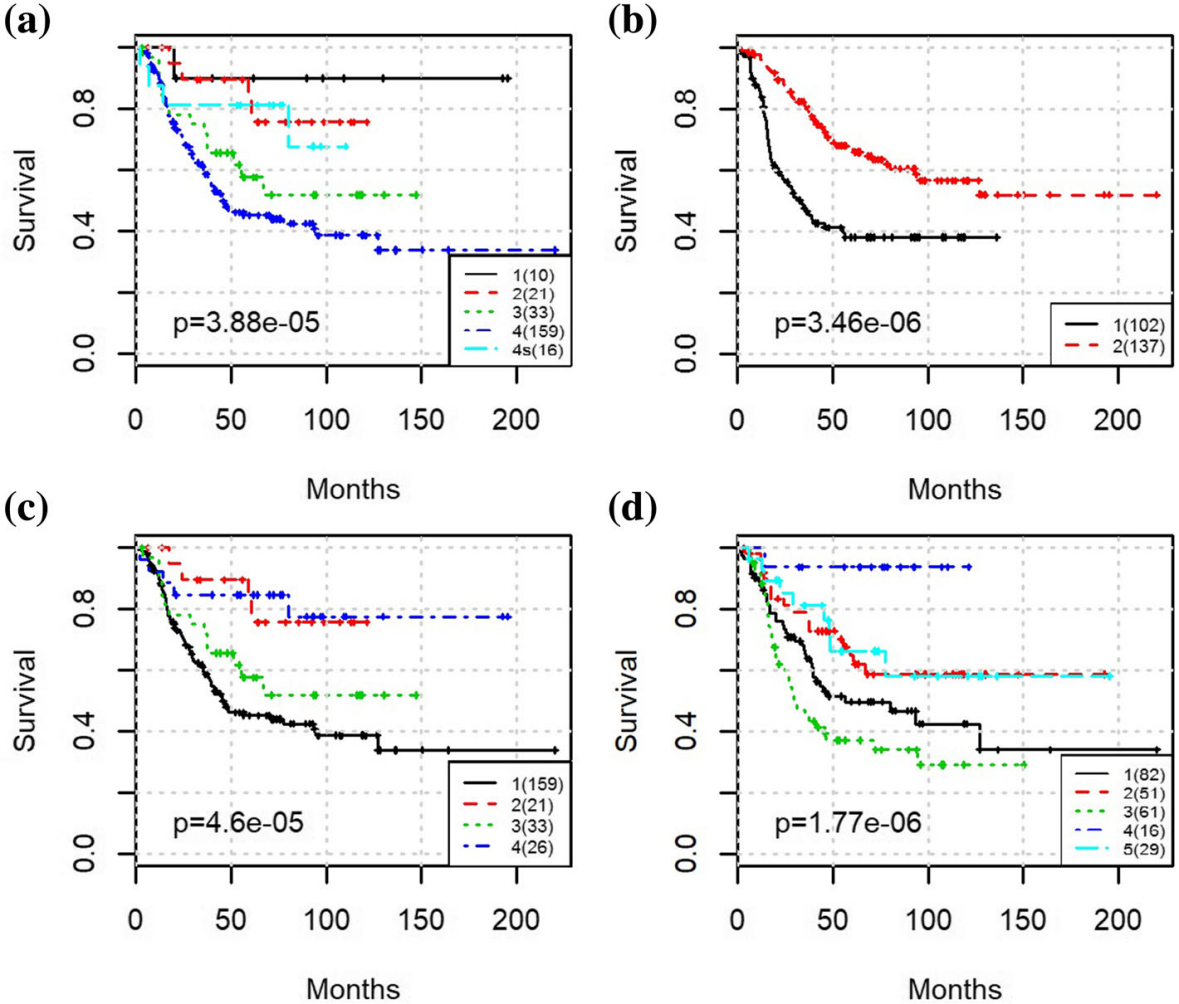
The Kaplan-Meier survival plot for the “high-risk” NB cohort in Fig. 4(c) cohort survival outcome among multiple methods. (a) Results from Clinical stage; (b) Results from SNF; (c) Results from MRCPS of scaled exponential similarity kernel integrated with clinical stage; (d) Results from MRCPS of molecular density kernel integrated with clinical stage

Interestingly, MRCPS using both molecular weight matrices identified a subgroup of 239 patients that has the significantly poorer survival rate of less than 40% at the end of the study (Fig. 3(c) Group 2&3, Fig. 4(c) Group 2&3). We noticed that in Fig. 4(d), the patients in Group 1 are all alive, and the clinical risk level also shows as low-risk level. This suggests that adding the transcriptomic features may improve the stratification for these “high risk” patients alone. By focusing on these 239 patients, we aimed to achieve better classification and identify the worse survival subgroup can be identified. After applying MRCPS with either of the two patient similarity matrix approaches on the poorer prognostic group of these 239 patients, an even higher risk subgroup was identified, and surprisingly, also a low-risk subgroup as well (Fig. 5). We then compared the clustering results by MRCPS and disease stage on these patients. These results are shown in Fig. 5. As aforementioned, although clinical features are capable of identifying the patients of low-risk subgroup, it does not further stratify the high-risk group with mixed outcomes very well (Fig. 5(a)). Figure 5(b) shows the clustering result of SNF using only the transcriptomic feature. K-means clustering (K = 2) generates the best clustering result with the maximal mutual information within each cluster. However, it is difficult to reconcile with the currently used five clinical stages. MRCPS with two patient similarity weight matrix generation approaches clustered these high-risk patients into four and subgroups respectively, as shown in Fig. 5(c) and (d). Figure 5(c) shows the clustering result of integrating patient similarity matrix with the scaled exponential similarity kernel approach. However, the log-rank *p* value is not better than the classification using clinical stages. In the Fig. 5(d), the results of MRCPS with density kernel showed the best prognosis performance (log-rank *p* = 1.77e-6), which still preserves five subgroups. We compared the good prognosis groups between the two approaches in Fig. 5(c) and (d). They are shown in the Additional file 3 and all the patients in group 4 in Fig. 5(d) are in either group 2 or group 4 in Fig. 5(c). More importantly, Fig. 5(d) results separated the majority of the stage IV patients into two groups, i.e., Group 1 and Group 3. It identified Group 3 with the worst prognosis, with less than 40% survived in the first 50 months of diagnosis.

**Fig. 6:**
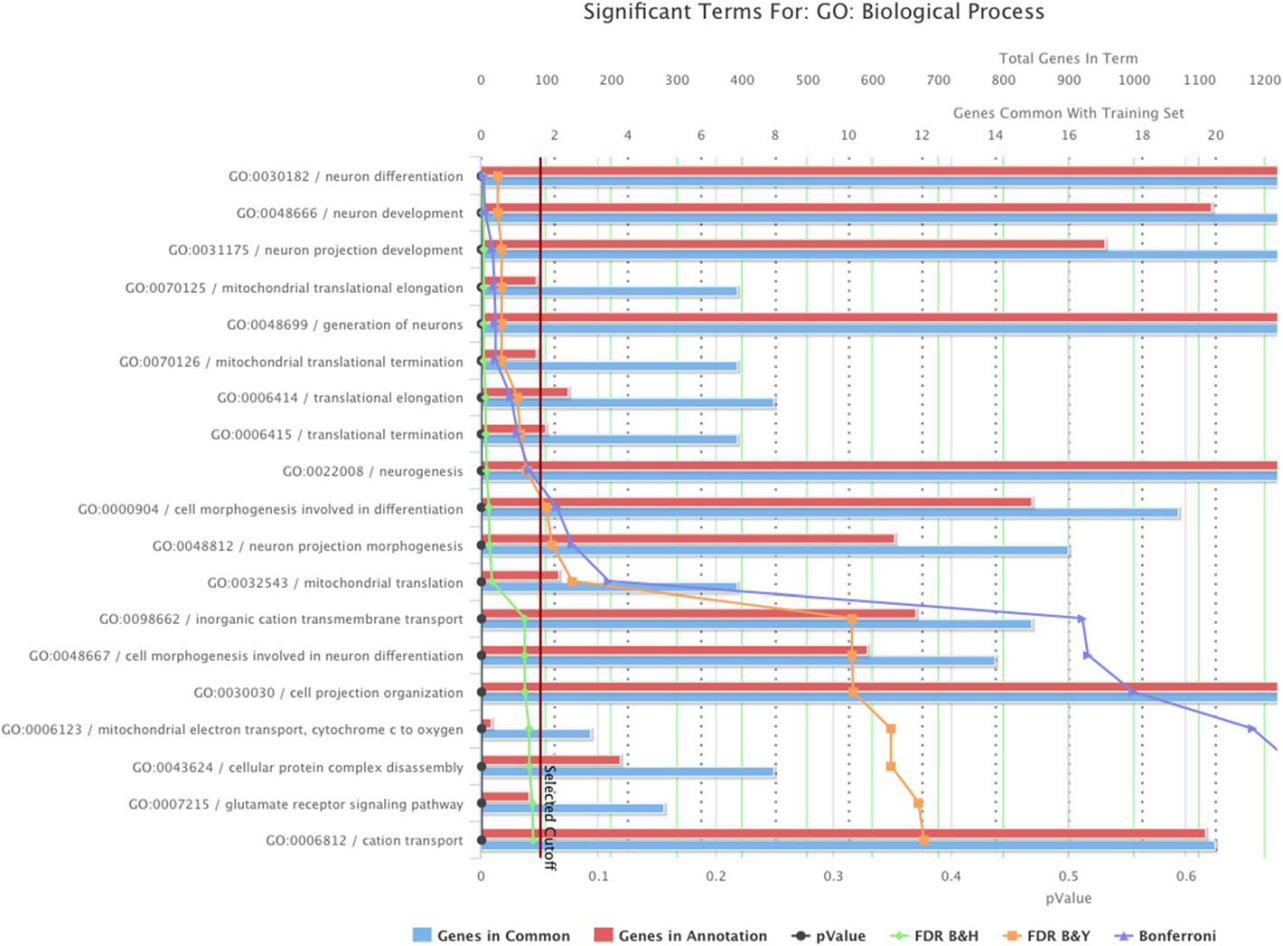
Gene ontology enrichment analysis using differentially expressed genes between patients in Group 4 (best prognosis) and Group 3 (worst prognosis) in Fig. 5(d)

We also identified highly differentially expressed genes between the patients in Group 4 (best prognosis) and Group 3 (worst prognosis) of Fig. 5(d) from RNA-seq data, then carried out the gene ontology over-representation analysis on the differentially expressed gene list. The results are shown in Fig. 6. All the top enriched biological processes are related to neuron differentiation and development, which fits this pediatric neurological disease context very well. The mitochondrial genes are also enriched, which suggests the energy production and metabolic pathways may play an role to differentiate the patients disease progression. These differentially expressed genes may harbor molecular level differences between the two prognostic groups, which can be potential gene biomarkers for clinical testing.

### The co-expression modules reveal genes previously associated with NB

From a parallel separate study where co-expression modules were further examined for their association with survival outcomes [17], we discovered that for co-expression modules from microarray data, the genes in Module 2, 7, 10, 36 and 37 are significantly associated with survival prognosis which shown in Additional file 4, and most genes are involved in cancer hallmark pathways. Specifically, Module 2 is highly enriched with cell cycle and cell division genes (97 out of total 127 genes, *p* = 1.45e-69). The genes in Module 7 are mostly involved in extracellular matrix organization (19/53, *p* = 3.88e-16) and angiogenesis (20/53, *p* = 1.12e-12). Module 10 is enriched with genes in immune response (16/42, *p* = 6.03e-4), angiogenesis (11/42, p = 6.03e-4), and extracellular component (15/42, *p* = 1.06e-4). Module 36 and 37 are also mostly immune response genes (4/10, *p* = 8.17e-7). All of above fit very well with the highly elevated biological processes in cancer cells. For co-expression modules from RNA-seq data, RNA-seq data Module 2,7, 17 and 21 are most significantly associated with survival outcome. RNA-seq data Module 2 includes most of the Module 2 genes from microarray data, which is enriched with the same cell cycle genes (144/268, *p* = 4.84e-73). RNA-seq data Module 17 and 21 are mostly zinc finger family proteins that play important roles in transcriptional regulation. The co-expressed module gene lists from microarray and RNA-seq data are shown in the Additional file 1.

We also crosschecked our gene co-expression module results with the genes previously known to be associated with NB. The microarray module 2 contains gene BIRC5, which previously found to be strongly overexpressed in neuroblastoma tumor samples and correlate to a poor prognosis, which could be a potential therapeutic target [9, 18]. Another study of NB [8] discovered that patients over one year of age with advanced stage and rapidly progressive disease generally have a near-diploid or near-tetraploid DNA karyotype and show recurrent segmental chromosomal copy number variations (CNVs), including allelic losses of 1p, 3p, 4p, 6q, 11q and 14q and gains of 1q, 2p and 17q. Study of [19] showing structural chromosomal abnormalities syntenic to segmental aberrations such as 17q gain, 2p gain and 1p36 LOH closely related to human MYCN-amplified NB. Among our co-expressed modules, module R13 all genes are located on 17q; R15 all genes are located on 1p36 1p36.33; R23 all genes are located on 3p; R24 all genes are located on 2q, which are consistent with the findings in [8] [19].

## Discussion and conclusion

In this paper, we modified the recently developed workflow MRCPS to integrate the transcriptomic data with the clinical features (clinical stage and clinical risk level) of NB patients. While the currently used clinical tumor stage can predict patient outcome reasonably well, it purely depends on the pathological features, which does not incorporate molecular features of the tumor, and fails to accurately identify the best and worst disease outcome patients from the high-risk group. Our integrative methods showed that this new workflow has superior performance to clinical staging for the NB cohort tested. MRCPS shows that “high-risk” group of patients can actually be further stratified into multiple groups with significantly different survival outcomes --- subgroups of patients with poor survival in early months were identified (Groups 1, 2, 3, and 5 in Fig. 5(d)), as well as a subgroup of high-risk patients has good prognosis (Group 4 in Fig. 5(d)). Further comparison of our stratification results with patient clinical stage information (Table 1) reveals an interesting finding: for the best survival group (Group 4) with 16 patients, 10 of them are from stage 2 patients while the rest six are all from stage 4 s patients, suggesting dramatic different outcomes exist even for the late stage patients. The analysis of differentially expressed genes between the refined best and worst prognostic groups indicates that the two subgroups contain genes behave differently in disease pathways, which is worth further investigation.

**Table 1:** The overall distribution of the patients in different stages in our stratification groups of Fig. 5(d)

	Stage 1 (*n* = 10)	Stage 2 (*n* = 21)	Stage 3 (*n* = 33)	Stage 4 s (*n* = 16)	Stage 4 (*n* = 159)
Group 1	0%	0%	0%	37.5%	47%
Group 2	60%	52%	100%	6%	0%
Group 3	30%	0%	0%	6%	37%
Group 4	0%	48%	0%	37.5%	0%
Group 5	10%	0%	0%	13%	16%

We also tested two types of patient similarity matrix constructions based on molecular features and found that MRCPS with density weight matrix method can stratify patients into robust and clinically relevant subtypes much better than the traditional tumor stage classification. MRCPS of scaled exponential similarity kernel method performs equally well in the entire cohort but not as good as the former in the high-risk cohort.

In summary, MRCPS consensus clustering workflow is a flexible workflow, allowing integration of both categorical and numerical data. The patient similarity matrix and molecular weighting schemes are adjustable. In the future, we will incorporate the genetic data (e.g., cope number variants and mutation data) with our current framework to improve the survival prognosis performance and verify our findings on other NB datasets.

## Reviewer comments

### Reviewer’s report 1: Lan Hu

1. Summarized that “This manuscript described a clean application of the authors’ original weighted network mining algorithm in NB patient gene expression data. The results showed that their approach improved prognosis significantly by clustering patients using the additional weighted similarity matrix information. Specifically, a subgroup of patients with extremely poor survival in early months was identified”

Author’s response: *We thank the reviewer for the encouraging comments on this work.*

2. “There are a few instances of placeholders in the manuscript that still remain to be filled with details. For example: in page 2, ‘the integrated workflow is shown in figure??’ Should fill in the figure number. In page 5, ‘the first is to use the original MRCPS algorithm to calculate the patient similarity matrix as described in section (Figure 3). The second approach is to use the message passing approach as described in section (Figure 4).’ What sections?”

Author’s response: *We have filled in all the placeholders with the corresponding figure and numbers, which are highlighted with yellow in the text. The sentences in page 5 were revised to "The first is to use the Cluster density function to calculate the patient similarity matrix (Figure 3), and the second approach is to use the scaled exponential sa “eigengene” > an ‘eigengene’ Molecular similar weight matrix > molecular.*

3. “Similarity matriximilarity kernel (Figure 4) as described in methods section.” on page 7

Author’s response: *We have corrected the first one as the reviewer suggested and highlighted it in the text. For the second one, we changed to “patient similarity matrix using molecular density function and the similarity network fusion method respectively” on page 4.*

4. “In Figure 1, spelling check for ‘molecular’ in page 6, ‘the clustering result of using molecular similarity weight matrix is worse than using the clinical stage, for molecular similar weight matrix using spectral clustering, we found that k = 2 is the best cluster result according to maximum mutual information, the result is shown in Figure 5(a), it is difficult to reconcile with the five clinical stages.’ Should break down into two sentences”

Author’s response: *We have corrected the above mistakes as the reviewer suggested and highlighted them in the text. The sentences in page 6 were revised to “Figure 5(b) shows the clustering result of SNF. k = 2 generates the best clustering result with the maximal mutual information within each cluster. However, it is difficult to reconcile with the currently used five clinical stages.”*

## Reviewer comments

### Reviewer’s report 2: Haibo Liu and Julie Zhu

1. Suggested to us that “This workflow could be useful for stratifying NB patients if the authors could validate its superiority with improved sensitivity and specificity by using independent data”

Author’s response: *We thank the reviewer for the very helpful suggestion for independent cohort validation, while this paper focuses on the dataset provided by the CAMDA contest, we are actively seeking additional validation dataset through the Pediatric Oncology program at Riley Children’s Hospital.*

2. “In addition, it would help readers to understand the algorithm better if the authors could give more detailed explanation to notations in formula (1), (5), (6) and (7)”

Author’s response: *We added the explanations for notations to the above four formulas to help readers understand them.*

3. “Formula (1) seems wrong since integration of this density function is not 1 over the sampling space. Also, based on the current definition, the formula (5) will always give 0. The formula should be corrected according to the original publication (cited by this paper as reference 1)”

Author’s response: *We corrected the formula.*

4. "Suggest authors do a spelling check and also make sure all figures are mentioned in the text. Here are a few examples. Page 1, Line 30, “build” should be “built”; “diagnose” should be “diagnosis”. The tense of verbs should be consistent in the abstract. Page 1, line 40, “neuroblastom survival time predict” should be “neuroblastom survival time prediction”; page 1, line 41, “consensus cluster” should be “consensus clustering”. Page2, Line31, what does the “??” stand for? Is it “1”? Similarly, some numbers are missing in page 5, lines 49 and 50, “section??”

Author’s response: *We thank the reviewer for the grammar and spelling corrections, we have corrected the such mistakes and highlighted them in the text. We also ran a thorough spelling check for the entire text.*

5. We recommend the authors search TCGA cBioPortal, we found there are at least 4 large scale studies of NB, with expression data and clinical data. The author should consider testing their methods on at least one of these datasets to show the reliability and superiority of their methods. Suggest the authors site the dataset used in this study, which is available in GEO and has been published by Zhang et al. 2015: https://genomebiology.biomedcentral.com/articles/10.1186/s13059-015-0694-1

Author’s response: *We thank the reviewers for their suggestions. In the manuscript, we actually used the same datasets as suggested by the reviewers in Zhang et. al publication. With the newly available datasets from TCGA, we plan to apply our workflow these datasets to validate our findings. We modified the description of the dataset used in this study and added reference of paper of Zhang et al. 2015.*

6. Suggest authors provide detailed information on processing of the microarray and RNA-seq data such as how batch effects were modeled. The authors should provide a brief description of how differential expression and gene ontology enrichment analysis were done in the method section, instead of putting it on page 18, lines 51–57

Author’s response: *We added the reference of raw data preprocessing and the section of the gene ontology and pathway enrichment analysis tool in the Methods section. As for the batch effect, we did the co-expression modules mining on gene pair correlation for RNA-seq and microarray dataset separately, not combined them together, and the expressions from each dataset was individually normalized then converted to z-score values, so any potential batch effect is removed. This pre-processing step was added in the Methods section. Differential expression analysis was added in the Method section with the foldchange cutoff 1.5 and adjusted p value cutoff of 0.001. Gene ontology enrichment analysis is also added in the Methods section.*

7. Why do the authors think that both microarray and RNA-seq data are needed for stratifying NB patients? Doesn’t RNA-seq providing more accurate measurement of gene expression? Do they suggest in the future researchers should acquire both types of expression data to better stratify NB patients? Some of the modules identified from co-expression analyses are very small, only contain a few genes. Are they stable clusters? Some of the clusters from RNA-seq and microarray assays overlaps to some degree, but many of them are so different. What’s the most important module for NB stratification? Perhaps validation with independent datasets will help to address this type of questions

Author’s response: *RNA-seq technique is the new transcriptomic quantification tool, which provide more details in gene expression than microarray technique, but a lot of transcriptomic analyses were done using microarray technique. In the manuscript we didn’t suggest researchers to obtain both types for their patient stratification. Instead, the reason we included both RNA-seq and microarray data for analysis is because we would like to investigate if the data type affects the co-expression mining result or not. We found that differences exist between the co-expression modules mined from microarray and RNA-seq data, which resulted in different patient classification results. In this study, we address the discrepancy by provide the flexible MRCPS method to incorporate the different co-exp results. We integrated the patients networks based on the different gene modules, and yield stable clusters. In a parallel study, we focused on the comparison these gene modules and the survival associated modules. The paper was accepted by Biology Direct will be publish soon. We added reference of this paper Result section.*

8. The explanation to the mathematic formulas could be improved. Since the methods are computationally intensive, to make their algorithms clear and reusable by other researchers, we strongly suggest the code/scripts be published along with the manuscript

Author’s response: *The first version of original MRCPS integration code is available in* *https://github.com/chaowang1010/MorCPS**. We are working on organizing the current version of code and uploading all parts of our pipeline together, it will be soon available on* *https://github.com/unicornH/MorCPS-2**.*

9. Language/writing can be further refined although it has been significantly improved in the revision. For example, the figure legend for Figures 2-4, “predict the entire NB cohort survival outcome …” is misleading. The survival outcomes of these patients are known instead of predicted, right? On page 18, line 24, need to add reference to “From separate studies …” . There are typos in the last box in the workflow, finial should be final

Author’s response: *We thank the reviewers to point out the typos and missing references. We have corrected them according to reviewer’s suggestions.*

10. Suggest authors review the latest advances of diagnosis, treatment and prognosis of NB in the introduction section, and compare their module genes to any genetic and molecular markers discovered so far in NB in the discussion section. It is important to discuss the results in the context of known biology of the NB. In the supplementary Table 1, the terms overrepresented among each module are displayed, which include chromosomal regions/cytoband. Has any of these regions been reported to be related to NB? Several recent reviews are suggested to be considered by the authors: http://www.cancerindex.org/geneweb/X1701.htm (1) https://academic.oup.com/jjco/article/48/3/214/4825045 (2) https://www.ncbi.nlm.nih.gov/pubmed/28055978 (3) https://www.ncbi.nlm.nih.gov/pubmed/29380702 (4)

Author’s response: *We thank the reviewer’s suggestion. We used gene set intersection between RNA-seq and microarray data in this paper, so it didn’t include all the genes mentioned in the above article. But we still found overlapping known NB genes as mentioned in above references. We added contents of comparing our identified module genes with the gene mentioned in these literatures in the Results section with the relevant references inserted.*

11. The supplementary tables lack of explanation. For example, there is no column name for Supp. Table 1. On page 33, a brief description of α and t would be helpful

Author’s response: *We added column name for Supplement Material 1. There are some typos regarding the parameters and their meaning. We clarified them in the manuscript Methods section. The two parameters t and determine an adaptive threshold of the density of the network, which the network mining algorithm will stop when the threshold is reached. The parameter alpha should be, previously mislabeled. We added the description of the parameter .*

## Reviewer comments

### Reviewer’s report 3: Aleksandra Gruca

1. “… Development of the methods for integration of heterogeneous data such as clinical information and transcriptomic experimental data allows not only validating and improving confidence in experimental results but also developing more complete more complete models of biological systems. In this context, the approach presented by the authors is very interesting, however, there are some issues in the paper that should be corrected in order to make its message more clear and understandable for the readers”

Author’s response: *We thank the reviewer for the comments of our methodology and fully agree to modify and clarify the text according to the reviewer’s suggestion so that it is more understandable to the readers.*

2. “The dataset is too briefly described … the data section should be expanded in order to provide the wider picture of the analysed dataset. In particular, there is no description of the clinical stage information (how it is defined? how many of them? how many patients are assigned to each clinical stage?)”

Author’s response: *We have added a detailed description of the transcriptomic dataset used in this study as well as the description on clinical stage information. They are highlighted with yellow in the text.*

3. The data used in this study is obtained from the neuroblastoma data integration challenge of CAMDA 2017. It contains tumor samples of 498 neuroblastoma patients from seven countries: Belgium (*n* = 1), Germany (*n* = 420), Israel (*n* = 11), Italy (*n* = 5), Spain (*n* = 14), United Kingdom (n = 5), and United States (n = 42). The patients’ age at diagnosis varied from 0 to 295.5 months (median age, 14.6 months)

Author’s response: *Transcriptome datasets from both microarray (Agilent Whole Human Genome 44 K Oligo microarray) and RNA-seq are obtained from the Neuroblastoma Data Integration Challenge of CAMDA 2017 for 498 pediatric patients with known clinical endpoints. The RNA-seq includes 60,788 transcripts and Agilent microarray data for 45,198 probes, both from 498 primary neuroblastomas. Tumor stage was classified according to the International Neuroblastoma Staging System (INSS): stage 1 (n = 121), stage 2 (n = 78), stage 3 (n = 63), stage 4 (n = 183), stage 4S (n = 53). 176 patients are labeled as high-risk, which the patients with stage 4 disease > 18 months at diagnosis and patients of any age and stage with MYCN-amplified tumors [13].*

4. “The middle step (transcriptomic data) clustering methods and the results are described very briefly. This part of the data processing should be presented in the paper in more detailed way. For example, the authors provide information that they were able to obtain 38 coexpressed gene modules for the mircoarray data and 24 modules for the RNAseq data. The information how the information from RNASeq experiment is integrated with the results of DNA microarray experiment is missing in the paper. The presentation of the result needs to be improved”

Author’s response: *We added this part in the Molecular Regularized Consensus Patient Stratification (MRCPS) section and used the formulas to explain how RNA-Seq and DNA microarray integrated together with two approaches.*

5. “There are some technical issues that should be corrected. First, there is no description of the legend for pictures. They are inconsistent with the description in the text (tumour stages 1,2,3,4 and 4s vs 1,2,3,4,5)”

Author’s response: *We thank the reviewer to point out the mistakes and added the description of the legend and corrected in the text as tumor stages 1,2,3,4 and 4 s.*

6. “the legend box covers the survival curves”

Author’s response: *We redrew the figure to fix this problem. The survival curves are not covered by legend box now.*

7. “Also clarify if the results presented in Fig 3a are based on k-means clustering (as in the figure description) or similarity network fusion algorithm (as in the text description)”

Author’s response: *We clarified the description, which highlighted in the text and the figure description. Figure 3(a) is from K-means clustering results.*

8. “Figures 3d and 4d present clustering results where clinical risk and clinical stage are integrated but in the methods part of the paper no explanation is provided how this two types of categorical data are combined”

Author’s response: *The L in the formula (12) is the set of clinical partitions of patients. The clinical risk level and clinical stage are integrated by using this formula. We added more description for this equation in the text.*

9. “Also, please explain why there are different numbers of groups for subfigures of Figures 3, 4 and 5. It is not clear from the paper how the number of clusters is determined”

Author’s response: *We added this part in the section of “Cluster number determination” in the revised version to explain how the number of clusters is determined. The result in Figures 3 and 4 are based on different patient similarity matrices. Figure 3 is based on MRCPS methods of molecular density. Figure 4 is based on MRCPS methods of scaled exponential similarity kernel. Therefore, they resulted in different clustering results, i.e. different number of groups. The results are explained in more details in the text.*

10. “The main deficiency of the paper is that the assessment of the presented framework is based only on survival analysis and pvalue statistics. Unfortunately, the authors do not try to provide any biological interpretation of the results presented on the figures”

Author’s response: *We thank the reviewer for this suggestion and added one more paragraph about the biological investigation of the co-expressed gene modules that are used to stratify patients. The following text are added to the Result section.*

*From separate studies where co-expression modules were further examined for their association with survival outcome, we discovered that for co-expression modules from Microarray data, The genes in Module 2, 7, 10, 36 and 37 are significantly associated with survival prognosis. Among them, Module 2 is highly enriched with cell cycle and cell division genes (97 out of total 127 genes, p = 1.45e-69), The genes in Module 7 are mostly involved in extracellular matrix organization (19/53, p = 3.88e-16) and angiogenesis (20/53, p = 1.12e-12). Module 10 is enriched with genes in immune response (16/42, p = 6.03e-4), angiogenesis (11/42, p = 6.03e-4), and extracellular component (15/42, p = 1.06e-4). Module 36 and 37 are also mostly immune response genes (4/10, p = 8.17e7). All of above fits very well with the highly elevated biological processes in cancer cells. For co-expression modules from RNA-seq data, The genes in Module 2,7, 17 and 21 are most significantly associated with survival outcome. Module 2 includes most of the Module 2 genes from microarray, and enriched with the same cell cycle genes (144/268, p = 4.84e-73). Module 17 and 21 are zinc finger family proteins that plays important roles in transcriptional regulation.*

*We also identified differentially expressed genes between the patients in Group 4 (best prognosis) and Group 3 (worst prognosis) of Figure 5(d), and carried out the gene ontology enrichment analysis using ToppGene (* *https://toppgene.cchmc.org/enrichment.jsp* *). The results are shown in Figure 6.*

11. “In particular, it is unknown how the new stratification groups are related to the original clinical clusters”

Author’s response: *The original clinical clusters are the clinical stages. The overall distribution of the patients in different stages in our stratification groups (generated using the density kernel MRCPS method and shown in Figure 5d) is shown in Table 1.*

12. “What are the groups 1 and 4 from fig 3(c) and how they are related to the groups 3 and 5 from the fig 4(d)? Please, explain.”

Author’s response: *There is no group 5 in the Figure 4(d), we think the reviewer meant Figure 3(d). The groups 1 and 4 from Figure 3(c) and groups 3 and 5 from the Figure 3(d) were obtained from MRCPS method. Figure 3 is based on the MRCPS of molecular density kernel and Figure 4 is based MRCPS methods of scaled exponential similarity kernel. There is substantial overlap between them: 84% Patients in group 3 and 5 from Figure 3(d) overlap with the patients in group 1 and 4 from Figure 3(c) and the details are shown in the Supplement Material 2.*

13. “Similar remarks concern the description of the results presented in figure 5.”

Author’s response: *The same situation is in Figure 5. They were from different MRCPS settings. We compared the good prognosis groups between the two approaches in Figure 5(c) and (d). They are shown in the Supplement Material 3 and all the patients in group 4 in Figure 5(d) are in either group 2 or group 4 in Figure 5(c).*

14. “There are some issues regarding indices in equation 5. Please check and correct accordingly”

Author’s response: *We corrected Equation 5.*

15. “Please, provide explanation what do you mean by clinical cluster”

Author’s response: *That is actually clinical stage, we corrected this description.*

16. "In the paper, the authors use several the expression “clinical features” or “clinical attributes” to describe division of papers to risk levels and clinical stage. Most people would assume that clinical features or attributes are related to additional medical information describing patients such as age, gender or any values that can result from medical examinations. To avoid confusion, please, state clearly in the introduction section of the paper what “clinical information” is and try to avoid using different expressions"

Author’s response: *We totally agree with the reviewer and further explained clinical feature as the clinical stage and risk level. We stick to clinical feature throughout the text.*

17. The methods used to obtain results that are mentioned in the “Biological evaluation of the co-expression modules” section should be described in methods section of the paper

Author’s response: *The method for co-expression module mining is lmQCM, which is explained in Methods section. The details of the module comparisons between microarray and RNA-seq data were further discussed in a separate publication. The paper was accepted by Biology Direct and will be publish soon. We added reference of this paper in the Results section.*

18. Also, in the “Biological evaluation of the co-expression modules” section, the authors mention several modules from gene expression data, but there are no such modules (2, 7, 10, 36 and 37) and its corresponding genes in the supplementary material 1

Author’s response: *We added the miss Modules to the Supplementary Material 4.*

19. The sentence starting from “Module 2 includes most of the Module 2 genes from microarray” is unclear. (the first mentioned module is from RNA-seq???). It is not always clear if the authors refer to the results from microarray data or from RNA-seq data

Author’s response: *We thank reviewer for the comments, we changed the sentence as “RNA-seq data Module 2 includes most of the Module 2 genes from microarray” to make it clearer.*

20. Results from supplementary material 1 should be presented more thoughtfully. The column B has no name. What is the meaning of ‘NS’. What is the meaning of the following notion (column B, row 26): GO:0006334 nucleosome assembly *p* = 1.925E-13; 6p22.1 *p* = 2.058E-6 (I might try to guess again but reading scientific results should not be about guessing)

Author’s response: *We added name for column B and modify the description so it can be better understood for the Supplementary Material 1.*

21. The English language in the manuscript is improved in comparison to the first version. However, still some corrections are needed. For example using plural/singular forms (Figures 2, Figures 3(a), module 2 gene, etc). Also the captions of the figures that starts with the word “predict” should be corrected, I assume it should be “prediction of” - please check carefully symbols in the text of the manuscript – they all should be in italic - supplementary materials 2 and 3 should be referenced in the text, not only in the response for reviewer’s comments

Author’s response: *We corrected these errors and inserted the supplementary Materials 2 and 3 reference in the text.*

22. Language of the manuscript still needs improvement. Please, prepare the final version with the help of native speaker (for example: module 2 gene are; module 2, 7, 10, 36 and 37 are significantly associated with survival prognosis which shown in supplement material 4)

Author’s response: *We thank the reviewer for the suggestion. We have edited the entire manuscript with the help of native English speaker.*

23. In supplement material 4, please delete headers of columns C up to end: “Co-expression Modules from RNAseq”

Author’s response: *we delete headers of columns C up to end: “Co-expression Modules from RNAseq”.*

### Reviewer’s report 4: Haibo Liu

1. Page 20, Lines 14–28, the authors mis-described their GO term and pathway analysis. What the authors did should be called “GO term and pathway over-representation analysis”, instead of “GO term and pathway enrichment analysis”. See papers https://journals.plos.org/ploscompbiol/article?id=10.1371/journal.pcbi.1002375 and https://www.ncbi.nlm.nih.gov/pmc/articles/PMC3829842/.

What was the background reference gene list used for over-representation analysis, whole genome or genes expressed in the target tumor tissue samples?

Author’s response: *We followed reviewer’s suggestion and used “over-representation analysis” instead of “enrichment analysis”. we used whole genome as background reference gene list. We clarified this in the method part.*

2. Page 20, Line 28, “Supplement Material 1 and 4” should be “Supplement Materials 1 and 4”. By the way, at the bottom of the table in the Supplement Material 4, the authors stated that pathway analysis was done using DAVID, instead as described in Lines 16–17. Please clarify.

Author’s response: *Thank the reviewer to point it out, we clarified them in the corresponding section*.

3. Page 20, Lines 32–41, the authors mentioned that Student t-test was used for RNA-seq differential expression analysis. Based on Methods, the FPKM values for RNA-seq gene expression were downloaded and used for further analysis. The FPKM values are not normally distributed, so t-test is not appropriate here. Log-transformation is needed before applying t-test. Notably, more recent comparative studies, such as https://academic.oup.com/bib/article/14/6/671/189645, indicate that FPKM normalization is not an appropriate normalization method for RNA-seq data analysis.

Author’s response: *We thank the reviewer for the very helpful suggestion, we used log-transformation before applying t-test, we clarified it and added this description in the method.*

4. Page 23, Lines 11–12, the authors stated that “we identified the same CNVs as the co-expression modules in our RNA module R13, R15, R23 and R24”. Throughout the manuscript, there is no other place where the authors mentioned CNV identification. So it is not reasonable to mention CNVs here.

Author’s response: *We delete this part according to reviewer’s suggestion.*

5.In the Result section of Abstract, Page 2 Lines 44–53, the authors list their results as: First, ….; secondly, ….; thirdly, … .. These three sentences should be rephrased to present results. Currently, those sentences are presenting methods.

Author’s response: *We thank the reviewer for the suggestion and rewrote the abstract.*

6.All “superior than” should be changed to “superior to”

Author’s response: *We changed it according to reviewer’s suggestion*.

7.Page 15, lines 9–11, “176 patients are labeled as high-risk, which are the patients with stage 4 disease of more than 18 months since diagnosis as well as patients of any age and stage with MYCN-amplified tumors [12]”. All “are” should be “were”

Author’s response: *We changed it according to reviewer’s suggestion.*

8.Page 15, line 58, “spectral cluster” should be “spectral clustering”. 5. Reference formats are not consistent

Author’s response: *We changed it according to reviewer’s suggestion.*

## Additional files

Additional file 1: Functional enrichment analysis for unique co-expression gene modules. (XLSX 18 kb)

Additional file 2: The overlapping ratio of the patients with good prognosis between the results from two MRCPS methos in Fig. 3. (XLS 19 kb)

Additional file 3: The overlapping ratio of the patients with good prognosis between the results from two MRCPS methos in Fig. 5. (XLS 19 kb)

Additional file 4: Functional enrichment analysis for the survival-associated co-expression gene modules. (XLSX 13 kb)

## Data Availability

The datasets are obtained from the Neuroblastoma Data Integration Challenge of CAMDA 2017.

## Abbreviations

GCN: Gene Co-expression Network
HR: High-risk
NB: Neuroblastoma
